# Analysis of cognitive function and its related factors after treatment in Meniere’s disease

**DOI:** 10.3389/fnins.2023.1137734

**Published:** 2023-04-04

**Authors:** Jiake Zhong, Xin Li, Jia Xu, Wenjing Chen, Juanjuan Gao, Xingxing Lu, Sichao Liang, Zhenping Guo, Manlin Lu, Yunshuo Li, Haijin Yi

**Affiliations:** Department of Otolaryngology, Head and Neck Surgery, Beijing Tsinghua Changgung Hospital, School of Clinical Medicine, Tsinghua University, Beijing, China

**Keywords:** Meniere’s disease, vertigo, vestibular dysfunction, cognitive decline, dizziness

## Abstract

A growing body of research recently suggested the association between vestibular dysfunction and cognitive impairment. Meniere’s disease (MD), a common clinical vestibular disorder, is usually accompanied by hearing loss and emotional stress, both of which may mediate the relationship between vestibule dysfunction and cognition. It is currently unknown whether the cognitive decline in MD patients could improve through treatment and how it relates to multiple clinical characteristics, particularly the severity of vertigo. Therefore, in the present study, the MD patients were followed up for 3, 6, and 12 months after treatment, and the cognitive functions, vertigo symptoms, and related physical, functional, and emotional effects of the patients were assessed using the Montreal Cognitive Assessment (MoCA) and Dizziness Handicap Inventory (DHI), aiming to explore the change in cognition before and after therapy and the correlation with various clinical features. It was found that cognitive decline in MD patients compared to healthy controls before therapy. Importantly, this cognitive impairment could improve after effective therapy, which was related to the severity of vertigo, especially in functional and physical impacts. Our results support the view that vestibular dysfunction is a potentially modifiable risk factor for cognitive decline.

## Introduction

Meniere’s disease (MD) is an idiopathic inner ear disorder characterized by recurrent spontaneous vertigo, low- to midfrequency sensorineural hearing loss, tinnitus, and/or aural fullness. The primary pathological feature is endolymphatic hydrops (EH; Lopez-Escamez et al., 2015). As one of the emergencies in clinical practice with increasing morbidity, MD not only limits activities of daily living (ADL) but also raises the risk of drop attacks. Recently, a growing body of findings suggested that patients with MD and other vestibulopathy may also experience cognitive decline, further decreasing quality of life and social functioning (Bigelow and Agrawal, 2015; Harun et al., 2015; Bigelow et al., 2016, 2020; Dobbels et al., 2019; Liu et al., 2019; Rizk et al., 2020; Chari et al., 2021; Dornhoffer et al., 2021; Bosmans et al., 2022). In fact, cognitive complaints in patients with vestibular symptoms are far from rare, even during vertigo intervals, they may suffer “brain fog” in the form of dullness, difficulty concentrating, poor memory or confusion (Bigelow et al., 2020; Chari et al., 2021). These complaints are more common in chronic vestibular syndromes such as MD and vestibular migraine (VM) compared to acute vestibular disorders like benign paroxysmal positional vertigo (BPPV; Liu et al., 2019; Rizk et al., 2020; Dornhoffer et al., 2021).

Although the association between vestibular dysfunction and cognitive impairment was gaining evidence, the picture became further complicated given that concomitant hearing loss has been independently recognized as a risk factor for dementia (Dobbels et al., 2019; Bosmans et al., 2020; Livingston et al., 2020). Furthermore, the unpredictability of MD attacks, chronic dizziness, and accompanying ear symptoms may increase mental stress to a certain extent in MD patients, such as anxiety, depression, panic, and dyssomnia. Those could mediate the relationship between vestibule dysfunction and cognition (Lahmann et al., 2015; Smith et al., 2019; Xie et al., 2022). However, existing studies on cognitive function in MD patients are quite limited and lack multidimensional longitudinal data. The correlation between cognitive performance and multiple clinical features of MD has not been clarified, and the question of whether cognitive decline could be preserved or improved after effective treatment remains poorly answered.

Therefore, we conducted a longitudinal prospective study, in which the MD patients were followed up for 3, 6, and 12 months after step therapy, aiming to explore the change in cognitive performance before and after treatment, and the correlation between this change with various clinical characteristics, particularly the severity of vertigo. Considering that vertigo, dizziness, imbalance, and cognitive symptoms are often vague and difficult to quantify, the Dizziness Handicap Inventory (DHI) and Montreal Cognitive Assessment (MoCA) were utilized in this study to describe them as comprehensively as feasible in terms of the cognitive functions, vertigo symptoms and related physical, functional, and emotional effects of the patients (Jacobson and Newman, 1990; Nasreddine et al., 2005; O’Driscoll and Shaikh, 2017; Jia et al., 2021). In addition, hearing status and degree of tinnitus were also included as vital variables for analysis. In line with this, the primary aim of this study is to evaluate the change in cognitive performance in MD patients after therapy and analyze the potentially related factors. The secondary purpose of this study is to examine the specific dimensions of cognitive decline in MD patients before treatment. It improves the reliability of the results by including a healthy control group and analyzing these results in comparison with the MD group.

## Subjects and methods

### Design

**Figure 1:**
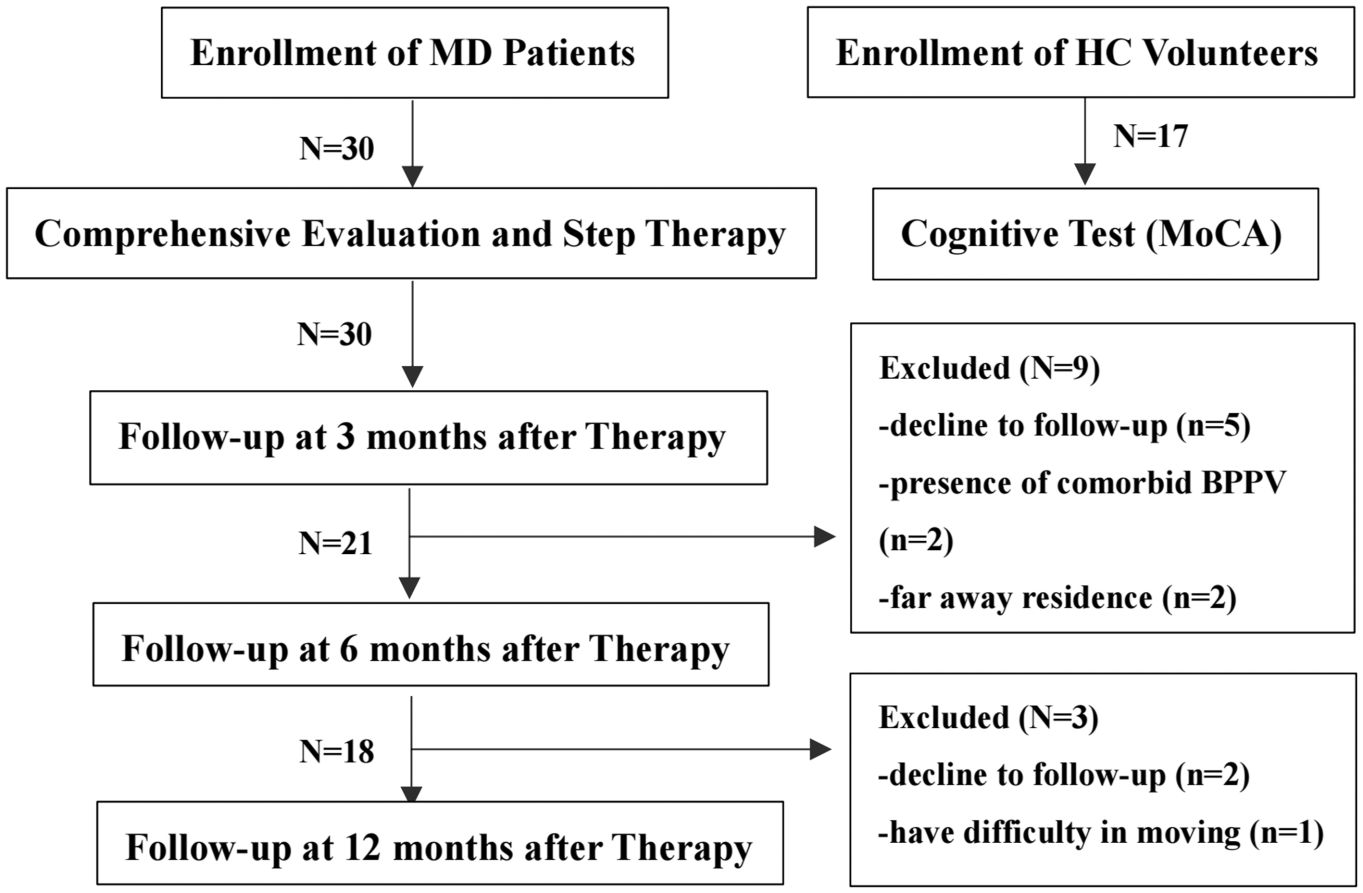
Study procedure and exclusion of participants. MD, meniere’s disease, HC, healthy controls; and MoCA, montreal cognitive assessment.

This is a single-center, longitudinal, prospective original study. Patients who were admitted to the Department of Otorhinolaryngology-Head and Neck Surgery at Beijing Tsinghua Changgung Hospital from November 2020 until April 2022 were recruited (MD group). Healthy Controls (HC group) were recruited through advertisements placed on boards in the hospital. The study procedure and the exclusion of participants for various reasons during follow-up were shown in the flow chart (Figure 1). The study protocol received ethical approval from our institutional review board (Approval Number: 23023-4-01). All participants provided written informed consent prior to enrollment.

### Subjects

For the MD group, patients with a diagnosis of MD according to the guideline formulated by the American Academy of Otolaryngology-Head and Neck Surgery (AAO-HNS; Basura et al., 2020) in 2020 were included. Meanwhile, we excluded patients: (1) with a history of external, middle, or other inner ear diseases or treatment; (2) with comorbid or alternative vestibular disorders such as BPPV, VM, or neurological disease; (3) with visual impairment that limited the cognitive tests; and (4) with other diseases or related family history that may interfere cognition function, such as cerebrovascular disease, Alzheimer’s disease (AD), schizophrenia, intracranial tumors, COVID-19, etc.

For the HC group, candidates with vertigo, dizziness, imbalance, hearing loss, tinnitus, or other MD symptoms, in addition to the above exclusion conditions, were excluded from the study. Two groups were matched in age, sex, and education.

### Intervention

All patients were administrated patient education and lifestyle adjustment (low-salt and low-fat diet, avoidance of tea and coffee, and sufficient sleep). A step therapy protocol was given to patients depending on the disease stage. The treatment approach started with oral medication, typically including betahistine mesylate (6–12 mg TID) and diuretics (hydrochlorothiazide, 25 mg BID). If symptoms persisted after the conservative treatment, patients were offered modified intratympanic steroid therapy (IST) by inserting a tympanic ventilation tube (VT) through an otoendoscope and placing gelatin sponges. Intratympanic injection (methylprednisolone, 40 mg) was carried out twice a week for a total course of 2 weeks. Individuals were provided more aggressive treatment if IST did not work. Patients in stage 2 with intolerance of frequent and severe vertigo episodes, as well as those in stages 3–4, were offered modified endolymphatic sac decompression surgery (ESDS) which combines posterior tympanotomy with local steroids (Xu et al., 2020). During post-treatment follow-up, if patients had residual dizziness or heavy feelings that could not be relieved by sleep regulation, they would be given oral betahistine mesylate (6–12 mg TID for 1–2 weeks).

### Outcomes

A comprehensive assessment was performed for all patients before treatment and 3, 6, and 12 months after treatment. This evaluation included pure tone audiometry (PTA), MoCA, DHI, and degree of tinnitus. HC group were administrated cognitive tests (MoCA). All scales were conducted in the same, quiet, well-lit interview room under the guidance of the same trained professional.

Pure tone audiometry was carried out using Astera audiometry (Otometrics, Denmark) with standard headphones and inserted earphones. The low- to midfrequency (500, 1,000, and 2,000 Hz) air conduction threshold average in the affected ear was calculated at its worst level during the interictal period. The average hearing thresholds for Stages 1–4 were 25 (dB HL), 26–40 (dB HL), 41–70 (dB HL), and > 70 (dB HL), respectively.

The MoCA, revised by Nasreddine et al. (2005) in 2004, was used to assess cognitive function. It covers visuospatial and executive function (five points), naming (three points), memory (five points), attention (three points), calculation (three points), language (three points), abstraction (two points), and orientation (six points). There are eight cognitive domains, 14 questions, and 30 points overall. One point is added to the total score for education years ≤12 to correct for educational attainment bias. Total scores <26 are deemed cognitive decline. Total and subdomain scores were collected.

The DHI, developed by Jacobson and Newman (1990), was used to evaluate the severity of vertigo. There are 25 items including physical (P, seven items), emotional (E, nine items), and functional (F, nine items) dimensions with three response levels (none, sometimes, always, and on a score of 0, 2, and 4). Scores of 0–30, 31–60, and 61–100 represent mild, moderate, and severe handicaps, respectively. The higher the score, the more serious the subjective symptoms and the impairment of vertigo-related physical activity, emotional psychology, and daily social functioning. The total DHI score and its P, E, and F scores were included for analysis.

There are five levels of tinnitus frequency and how it affects the patient’s life: No tinnitus in class 0; Class 1, infrequent (intermittent) tinnitus that has no negative effects on sleep or productivity; Class 2, chronic tinnitus during quiet periods, which does not disrupt sleep; Class 3, chronic tinnitus that interferes with sleep; Class 4, persistent tinnitus that interferes with sleep and work; and Class 5, unbearable, persistently severe tinnitus (Feldmann, 1992).

### Statistics

Statistical analysis of data was performed using SPSS (IBM 26.0, Armonk, NY, United States). Normality was determined by the Shapiro–Wilk test and visualizing data in histograms and Q-Q plots. Continuous variables were expressed as mean ± SD and median [interquartile range (IQR)], while categorical ones were presented as frequency (percentage). Group comparisons (MD vs. HC, Pre-and Post-treatment in MD) were studied using the Independent-samples *t*-test and Paired-samples *t*-test for normally distributed variables, and Mann Whitney-U test and Wilcoxon signed-rank test for non-parametric data. Improvements were defined as the subtraction of pre-and post-treatment scores or levels. Pearson’s and Spearman’s rank correlation tests were used to analyze the degree and significance of the association between improvements in cognitive tests and clinical features. Multivariate analyses were studied using linear regression with the continuous variables of cognitive improvements as dependent variables, and age, gender, education, duration of disease, and changes in clinical characteristics as independent variables. The residuals, homoscedasticity, and independence were examined to ensure they fulfilled all linear regression assumptions. Effect sizes (Cohen *d*) and power analysis were presented using G*Power statistical software version 3.1, with *d* of 0.2 indicating a small effect; 0.5, a medium effect; and 0.8, a large effect. For all tests, statistical significance was set at *p* < 0.05.

## Results

### Demographical and clinical properties of the participants

A total number of 30 patients, including 21 females and nine males, were enrolled in the study. The age of the subjects ranged from 26 to 73 years, with a median age of 61.5 (20.0) years. The mean/median years of education and duration of disease were 12.18 ± 5.05 and 2.04 (6.0) years, respectively. All patients were diagnosed with definite MD and received step therapy.

A total number of 17 HC were recruited, including eight females and nine males. The age of HC ranged from 29 to 76 years, with a median age of 59.0 (22.0) years and the mean years of education were 11.27 ± 3.88 years. There were no statistical differences between MD patients and HC regarding age, gender, and education (*p* > 0.05). The demographical and clinical properties of all participants are presented in Table 1.

**Table 1:** Demographical and clinical properties of the participants.

Characteristic		MD (*N* = 30)	HC (*N* = 17)	*p* value
Age	median (IQR), y	61.5 (20.0)	59.0 (22.0)	0.974^a^
Gender	*N* (%)			0.109^b^
	Male	9 (30.0)	9 (52.9)	
	Female	21 (70.0)	8 (47.1)	
Laterality	*N* (%)		-	-
	Left	16 (53.3)		
	Right	12 (40.0)		
	Bilateral	2 (6.7)		
Education	Mean ± SD, y	12.18 ± 5.05	11.27 ± 3.88	0.654^c^
Duration of disease	Median (IQR), y	2.04 (6.0)	-	-
Disease Stage	*N* (%)		-	-
	Stage 1	4 (13.3)		
	Stage 2	3 (10.0)		
	Stage 3	17 (56.7)		
	Stage 4	6 (20.0)		
Treatment	*N* (%)		-	-
	Oral drug therapy	6 (20.0)		
	IST	17 (56.7)		
	ESDS	7 (23.3)		

MD, meniere’s disease; HC, healthy controls; IQR, interquartile range; SD, standard deviation, ^a^Mann Whitney-U test, ^b^Pearson Chi-Square test, and ^c^Independent-samples *t*-test.

### MoCA scores were significantly lower in MD compared with HC

There was a significant difference between MD and HC in MoCA total scores (22.63 ± 4.06 vs. 25.12 ± 2.74, *p* < 0.05, Cohen *d* = 0.72). Concerning the subdomains, Memory scores in MD were significantly lower than in HC (*p* < 0.01, Cohen *d* = 0.88). However, there was no significant difference between the groups regarding other subdomain scores (*p* > 0.05), as shown in Table 2. Since Cohen’s *d* effect sizes of group comparisons in the Visuospatial/Executive, Attention, and Orientation scores showed a medium effect, combined with the power analysis (Power = 0.41, 0.64, and 0.53), the *p* > 0.05 might be the result of limited sample sizes.

**Table 2:** Comparison of MoCA scores for MD and HC.

MoCA scores	MD (*N* = 30)	HC (*N* = 17)	*t/Z*	*p* value	Cohen *d*
Total scores	22.63 ± 4.06	25.12 ± 2.74	2.496^a^	^*^0.016	**0.72**
Visuospatial/Executive	3.0 (2.0)	4.0 (1.0)	1.275^b^	0.202	**0.45**
Naming	3.0 (0.0)	3.0 (1.0)	0.045^b^	0.964	<0.1
Memory	1.0 (3.0)	4.0 (2.0)	2.643^b^	^**^0.008	**0.88**
Attention	3.0 (1.0)	3.0 (0.0)	1.863^b^	0.062	**0.63**
Language	2.0 (2.0)	2.0 (2.0)	0.495^b^	0.620	0.15
Calculation	3.0 (0.0)	3.0 (0.0)	0.549^b^	0.583	<0.1
Abstraction	1.0 (1.0)	1.0 (1.0)	0.311^b^	0.756	<0.1
Orientation	6.0 (1.0)	6.0 (0.0)	1.734^b^	0.083	**0.54**

The bold values mean Cohen *d* ≥ 0.45. MoCA, montreal cognitive assessment; MD, meniere’s disease; HC, healthy controls. ^a^Independent- samples *t*-test, ^b^Mann Whitney-U test, ^*^p < 0.05, ^**^p < 0.01.

### Treatment outcomes of the patients

At 3, 6, and 12 months post-treatment, 30, 21, and 18 individuals received follow-up in total. Statistical analysis demonstrated the change in clinical characteristics before and after treatment, including the severity of vertigo, hearing thresholds, and degree of tinnitus, as indicated in Tables 3, 4. The total DHI score and its physical, emotional, and functional scores were significantly lower after treatment, with *p* < 0.0001. There were statistical differences in hearing thresholds at 3 and 6 months post-treatment (*p* < 0.05) with an average decrease of 5.83 (20.0) and 6.67 (18.34; dB HL), while there was no obvious difference at 12 months post-treatment (*p* > 0.05). Concerning the degree of tinnitus, there were statistically significant differences between pre-treatment and 3 and 6 months post-treatment (*p* < 0.05) but no significant difference at 12 months post-treatment (*p* > 0.05).

**Table 3:** Comparison of DHI and PTA pre- and post-treatment for MD patients.

	Pre-treatment	Post-treatment	*t/Z*	*p* value	Cohen *d*
3-month posttreatment *N* = 30					
DHI	60.0 (20.0)	9.0 (39.0)	8.185^a^	^**^0.000	**1.97**
DHI-P	23.0 (11.0)	5.0 (15.0)	6.755^a^	^**^0.000	**1.66**
DHI-E	13.0 (13.0)	1.0 (10.0)	5.620^a^	^**^0.000	**1.24**
DHI-F	28.0 (12.0)	4.0 (21.0)	4.426^b^	^**^0.000	**1.85**
PTA	53.8 ± 22.2	46.1 ± 26.7	2.424^b^	^*^0.015	0.32
6-month posttreatment *N* = 21					
DHI	62.0 (14.0)	4.0 (35.0)	3.885^b^	^**^0.000	**2.25**
DHI-P	24.0 (8.0)	2.0 (14.0)	5.899^a^	^**^0.000	**1.70**
DHI-E	12.0 (11.0)	0.0 (6.0)	8.154^a^	^**^0.000	**1.71**
DHI-F	30.0 (9.0)	2.0 (16.0)	8.024^a^	^**^0.000	**2.26**
PTA	54.7 ± 22.2	46.7 ± 26.9	2.129^b^	^*^0.033	0.33
12-month post-treatment *N* = 18					
DHI	62.0 (21.0)	9.0 (40.0)	6.938^a^	^**^0.000	**2.08**
DHI-P	24.0 (8.5)	3.0 (15.0)	5.434^a^	^**^0.000	**1.75**
DHI-E	12.0 (11.0)	1.0 (7.0)	4.482^a^	^**^0.000	**1.44**
DHI-F	29.0 (10.0)	2.0 (13.0)	7.812^a^	^**^0.000	**2.12**
PTA	49.7 ± 23.6	45.8 ± 27.6	0.963^a^	0.349	0.15

The bold values mean Cohen *d* ≥ 0.45. DHI, dizziness handicap inventory; DHI-P, DHI-physical scores; DHI-E, DHI-emotional scores, DHI-F, DHI-functional scores; PTA, pure tone average; ^a^Paired-samples *t*-test, ^b^Wilcoxon signed-rank test, ^*^p < 0.05, and ^**^p<0.01.

**Table 4:** Comparison of degree of tinnitus pre- and post-treatment for MD patients.

Tinnitus	Pre-treatment	Post-treatment	*Z*	*p* value
3-month post-treatment *N* = 30				
Class 0	1 (3.3%)	9 (30%)	3.471^a^	^**^0.001
Class 1	5 (16.7%)	7 (23.3%)		
Class 2	18 (60.0%)	12 (40.0%)		
Class 3	4 (13.3%)	2 (6.7%)		
Class 4	2 (6.7%)	0 (0.0%)		
6-month post-treatment *N* = 21				
Class 0	1 (4.8%)	4 (19.0%)	2.506^a^	^*^0.012
Class 1	4 (19.0%)	7 (33.3%)		
Class 2	11 (52.4%)	8 (38.1%)		
Class 3	3 (14.3%)	1 (4.8%)		
Class 4	2 (9.5%)	1 (4.8%)		
12-month post-treatment *N* = 18				
Class 0	1 (5.6%)	4 (22.2%)	1.563^a^	0.118
Class 1	5 (27.8%)	4 (22.2%)		
Class 2	9 (50.0%)	9 (50.0%)		
Class 3	2 (11.1%)	1 (5.6%)		
Class 4	1 (5.6%)	0 (0.0%)		

a Wilcoxon signed-rank test.

^*^p < 0.05, ^**^p < 0.01.

### The pre-treatment and post-treatment MoCA scores were significantly different

**Figure 2:**
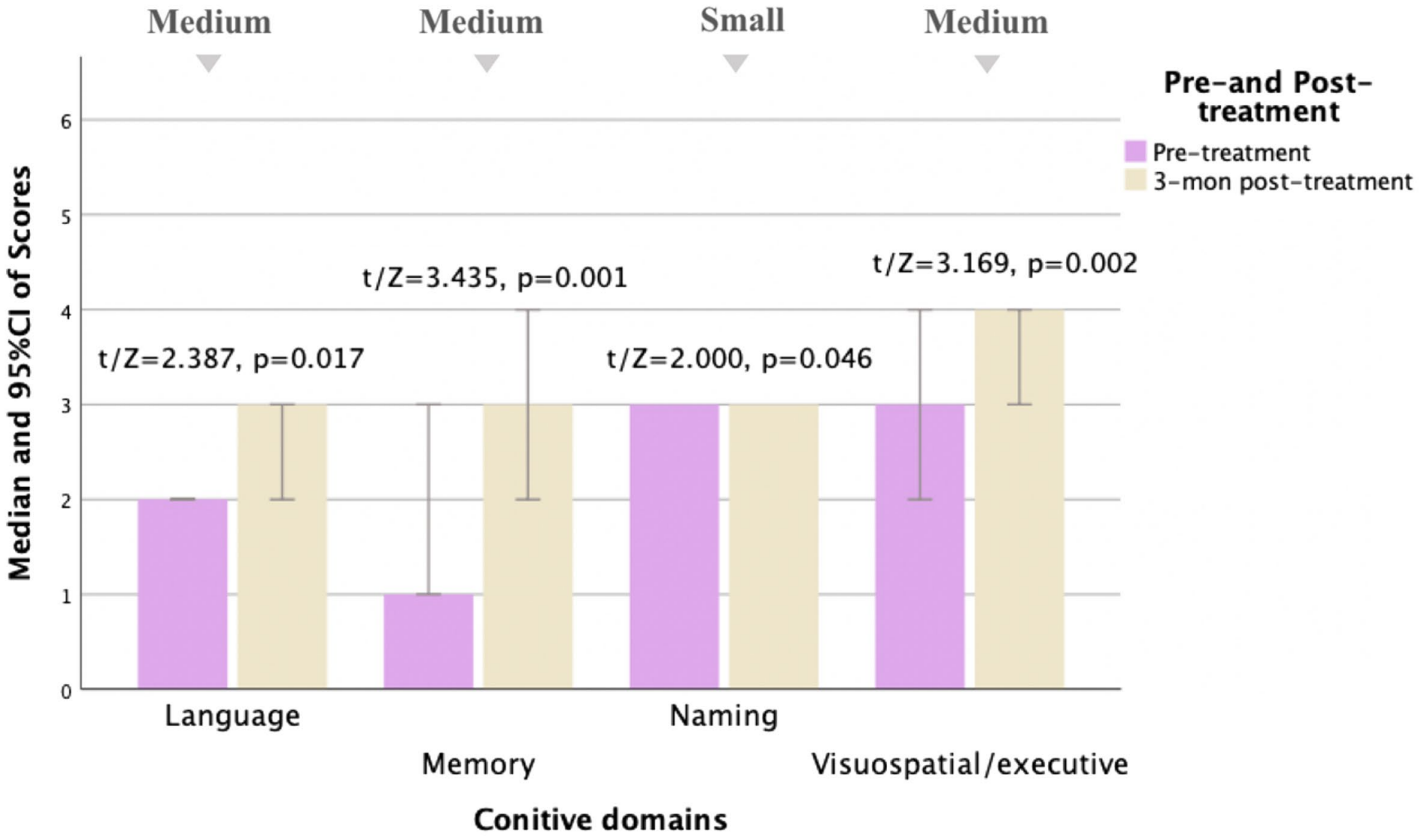
Comparison of MoCA subscale scores pre- and 3 months post-treatment for MD patients (*N* = 30). Compared to pre-treatment, MoCA subscale scores improved mainly in language, memory, naming, and visuospatial/executive aspects (*t*/Z = 2.387, 3.435, 2.000, and 3.169, and *p* < 0.05) at 3 months post-treatment, especially in memory and visuospatial/executive aspects (*p* < 0.01). Small, medium, and large indicate clinically meaningful Cohen *d* effect sizes.

**Figure 3:**
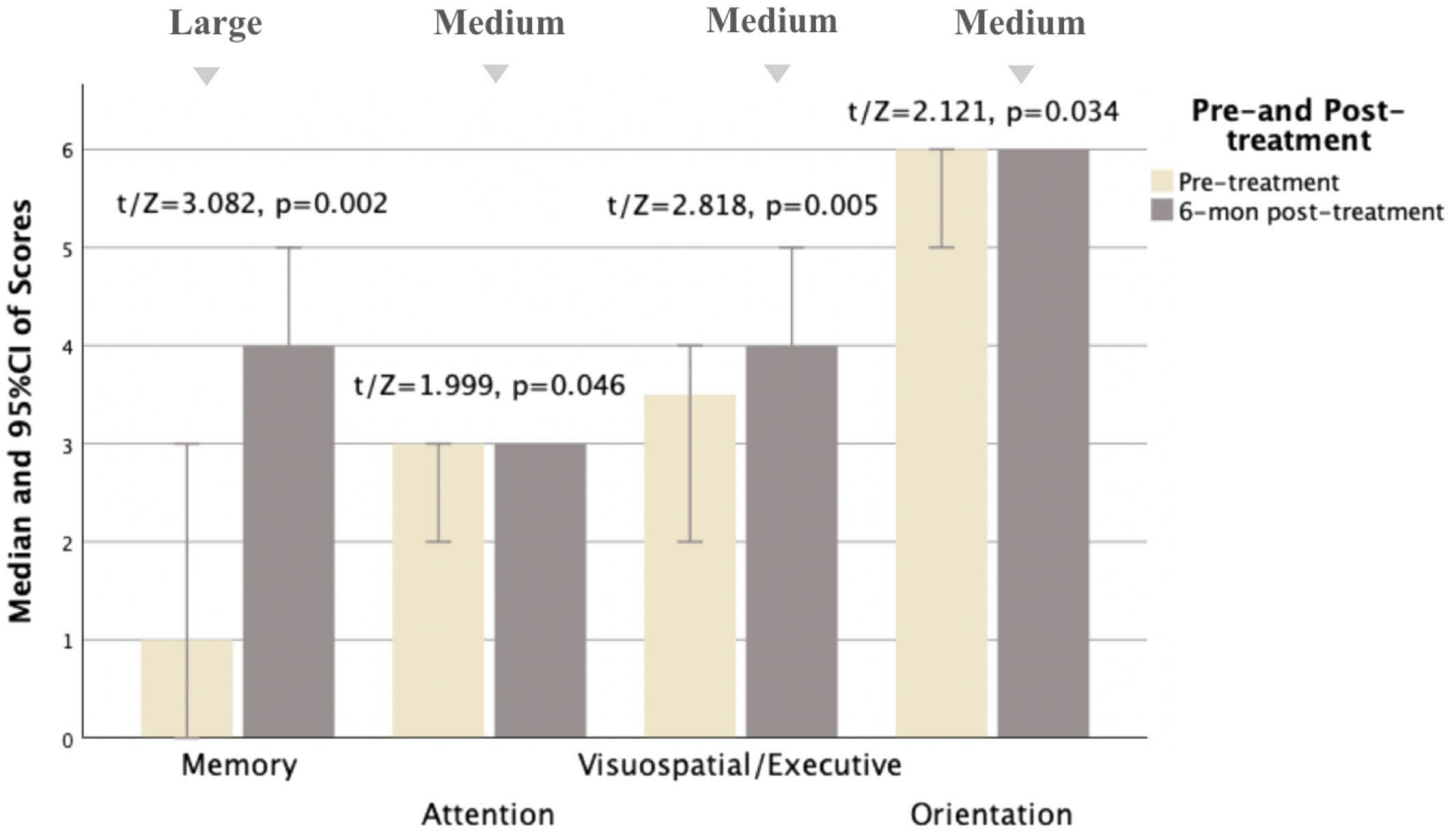
Comparison of MoCA subscale scores pre- and 6 months post-treatment for MD patients (*N* = 21). Compared to pre-treatment, MoCA subscale scores improved mainly in memory, attention, visuospatial/executive, and orientation aspects (*t*/Z = 3.082, 1.999, 2.818, and 2.121, and *p* < 0.05) at 6 months post-treatment, especially in memory and visuospatial/executive aspects (*p* < 0.01). Small, medium, and large indicate clinically meaningful Cohen *d* effect sizes.

**Figure 4:**
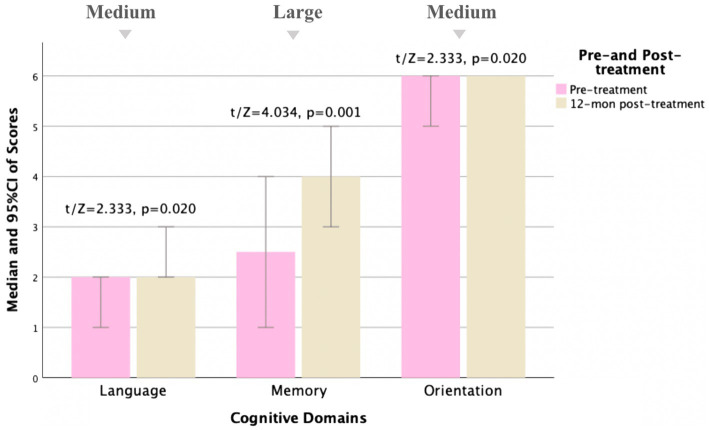
Comparison of MoCA subscale scores pre- and 12 months post-treatment for MD patients (*N* = 18). Compared to pre-treatment, MoCA subscale scores improved mainly in language, memory, and orientation aspects (*t*/Z = 2.333, 4.034, and 2.333, and *p* < 0.05) at 12 months post-treatment, especially in memory aspects (*p* < 0.01). Small, medium, and large indicate clinically meaningful Cohen *d* effect sizes.

The MoCA was performed before and 3, 6, and 12 months after treatment, respectively. The pre-treatment and post-treatment MoCA scores in total and subdomains were compared (Table 5). Concerning the total MoCA scores, the median increased from 23.0 (8.0) to 26.0 (4.0) at 3 months post-treatment (Cohen *d* = 0.64, Power = 0.92), from 24.0 (9.0) to 27.0 (6.0) at 6 months post-treatment (Cohen *d* = 0.74, Power = 0.88), and from 23.0 (9.0) to 27.5 (7.0) at 12 months post-treatment (Cohen *d* = 0.77, Power = 0.87), with significant differences (*p* < 0.0001). As detailed in Table 5, of the subdomains, the visuospatial and executive function and memory were significantly improved after treatment (Cohen *d* > 0.5, *p* < 0.01). Furthermore, the orientation, attention, and language were also improved, with Cohen *d* > 0.5, *p* < 0.05, as shown in Figures 2–4.

**Table 5:** Comparison of MoCA pre- and post-treatment for MD patients.

MoCA	Pre-treatment	Post-treatment	*t/Z*	*p* value	Cohen *d*
3-month post-treatment *N* = 30					
Total scores	23.0 (8.0)	26.0 (4.0)	4.889^a^	^**^0.000	**0.64**
Visuospatial/Executive	3.0 (2.0)	4.0 (2.0)	3.169^b^	^**^0.002	**0.55**
Naming	3.0 (0.0)	3.0 (0.0)	2.000^b^	^*^0.046	0.29
Memory	1.0 (3.0)	3.0 (2.0)	3.435^b^	^**^0.001	**0.64**
Attention	3.0 (1.0)	3.0 (0.0)	1.485^b^	0.138	0.35
Language	2.0 (2.0)	3.0 (1.0)	2.387^b^	^*^0.017	**0.53**
Calculation	3.0 (0.0)	3.0 (0.0)	1.890^b^	0.059	0.29
Abstraction	1.0 (1.0)	2.0 (1.0)	1.291^b^	0.197	0.24
Orientation	6.0 (1.0)	6.0 (0.0)	0.000^b^	1.000	<0.1
6-month post-treatment *N* = 21					
Total scores	24.0 (9.0)	27.0 (6.0)	3.837^b^	^**^0.000	**0.74**
Visuospatial/Executive	3.0 (2.0)	4.0 (2.0)	2.818^b^	^**^0.005	**0.56**
Naming	3.0 (0.0)	3.0 (1.0)	0.000^b^	1.000	<0.1
Memory	1.0 (4.0)	4.0 (4.0)	3.082^b^	^**^0.002	**0.80**
Attention	3.0 (1.0)	3.0 (1.0)	1.999^b^	^*^0.046	**0.55**
Language	2.0 (2.0)	2.0 (1.0)	1.811^b^	0.070	0.42
Calculation	3.0 (0.0)	3.0 (0.0)	0.378^b^	0.705	<0.1
Abstraction	1.0 (1.0)	2.0 (1.0)	1.890^b^	0.059	0.37
Orientation	6.0 (1.0)	6.0 (0.0)	2.121^b^	^*^0.034	**0.68**
12-month post-treatment *N* = 18					
Total scores	23.0 (9.0)	27.5 (7.0)	4.699^a^	^**^0.000	**0.77**
Visuospatial/Executive	4.0 (2.0)	5.0 (2.0)	1.408^b^	0.159	0.32
Naming	3.0 (0.0)	3.0 (0.0)	1.414^b^	0.157	0.25
Memory	2.5 (3.3)	4.0 (2.0)	4.034^a^	^**^0.001	**0.87**
Attention	3.0 (1.0)	3.0 (1.0)	0.791^b^	0.429	0.28
Language	2.0 (1.0)	2.0 (1.0)	2.333^b^	^*^0.020	**0.51**
Calculation	3.0 (0.0)	3.0 (0.0)	1.069^b^	0.285	0.37
Abstraction	1.0 (1.0)	2.0 (1.0)	2.333^b^	0.157	0.35
Orientation	6.0 (1.0)	6.0 (0.0)	2.333^b^	^*^0.020	**0.65**

The bold values mean Cohen *d* ≥ 0.45. MoCA, montreal cognitive assessment, ^a^Paired-samples *t*-test, ^b^Wilcoxon signed-rank test, ^*^p < 0.05, ^**^p < 0.01.

### Reduced severity of vertigo was correlated with cognitive improvement

Correlation analysis suggested that the increase in MoCA scores was positively associated with the decrease in DHI and its physical and functional scores at 3 months post-treatment (*r/r_s_* = 0.512, 0.446, 0.496, and *p* < 0.05) and in DHI and its Physical scores at 12 months post-treatment (*r/r_s_* = 0.561, 0.584, and *p* < 0.05). No significant association were noted between cognitive improvements and the change in DHI-Emotional, DHI-Physical (6 months post-treatment), DHI-Functional (12 months post-treatment), hearing thresholds, or tinnitus degrees (*p* > 0.05), as illustrated in Table 6.

**Table 6:** Correlation of improvements in MoCA scores and clinical characteristics.

Changes in clinical characteristics	MoCA improvements					
	3-month *N* = 30		6-month *N* = 21		12-month *N* = 18	
	*r/r_s_*	*p* value	*r/r_s_*	*p* value	*r/r_s_*	*p* value
DHI total	0.512^a^	^**^0.004	0.079^b^	0.732	0.561^a^	^*^0.016
DHI-Physical	0.446^a^	^*^0.014	0.095^b^	0.682	0.584^a^	^*^0.011
DHI-Emotional	0.342^a^	0.064	0.016^b^	0.944	0.405^a^	0.095
DHI-Functional	0.496^b^	^**^0.005	0.102^b^	0.660	0.452^a^	0.060
Hearing Thresholds	0.180^b^	0.342	0.118^b^	0.609	0.009^a^	0.970
Tinnitus Degrees	0.130^b^	0.494	0.179^b^	0.437	0.253 ^b^	0.311

DHI, dizziness handicap inventory; MoCA, montreal cognitive assessment; ^a^Pearson’s correlation test; ^b^Spearman’s rank correlation test; ^*^p < 0.05, ^**^p < 0.01.

In multivariate analyses, the changes in DHI (*β* = −0.055, 95% CI [−0.093, −0.018] at 3 months post-treatment, and *β* = −0.087, 95% CI [−0.144, −0.030] at 12 months post-treatment), DHI-Functional (*β* = −0.102, 95%CI [−0.180, −0.024] at 3 months post-treatment), and DHI-Physical scores (*β* = −0.158, 95%CI [−0.604, 0.040] at 12 months post-treatment) were associated with improvements in MoCA scores over time (*p* < 0.05), as indicated in Table 7. But baseline age, education, duration of disease, and gender had no obvious correlation to cognitive improvements (*p* > 0.05).

**Table 7:** Regression analyses of improvements in MoCA scores and multi-factors.

Variables	β	95% CI	*p* value	*p*	*R^2^*
3-month post-treatment *N* = 30					
Age	0.031	[−0.048, 0.110]	0.424	^*^0.049^a^	0.171
Education	0.113	[−0.111, 0.338]	0.308		
DHI-Functional	−0.102	[−0.180, −0.024]	^*^0.012		
3-month post-treatment *N* = 30					
Age	0.036	[−0.042, 0.114]	0.353	^*^0.048^a^	0.199
Education	0.118	[−0.111, 0.347]	0.298		
Duration of disease	0.054	[−0.122, 0.230]	0.531		
DHI	−0.055	[−0.093, −0.018]	^**^0.005		
12-month post-treatment *N* = 18					
Age	−0.007	[−0.120, 0.105]	0.892	^*^0.041^a^	0.361
Education	−0.227	[−0.532, 0.078]	0.132		
Duration of disease	−0.282	[−0.280, −0.036]	0.081		
DHI-Physical	−0.158	[−0.604, 0.040]	^*^0.015		
12-month post-treatment *N* = 18					
Age	−0.041	[−0.157, 0.074]	0.449	^*^0.034^a^	0.426
Education	−0.251	[−0.544, 0.042]	0.087		
Duration of disease	−0.135	[−0.478, 0.207]	0.406		
Gender	2.621	[−0.409, 5.651]	0.084		
DHI	−0.087	[−0.144, −0.030]	^**^0.006		

DHI, dizziness handicap inventory; MoCA, montreal cognitive assessment; ^a^Multiple linear regression. Due to collinearity, DHI, DHI-Physical, and DHI-Functional scores were modeled individually for calculation. Dependent variables: Improvements in MoCA scores. Independent variables: age, education, gender, duration of disease, DHI, DHI-Physical, and DHI-Functional scores. β, regression coefficient; CI, confidence interval; *p*, *p* value for the regression model; and *R*^2^, coefficient of determination, ^*^p < 0.05, ^**^p < 0.01.

## Discussion

In recent years, there has been evolving evidence of the association between cognitive impairment and vestibular dysfunction, especially common in chronic vestibular syndromes, such as MD (Hitier et al., 2014; Bigelow and Agrawal, 2015; Harun et al., 2015; Aitken et al., 2016; Bigelow et al., 2016, 2020; Benoit et al., 2018; Dobbels et al., 2019; Liu et al., 2019; Rizk et al., 2020; Chari et al., 2021; Dornhoffer et al., 2021; Bosmans et al., 2022). Various studies have directly or indirectly demonstrated the presence of cognitive dysfunction in MD patients. Seo et al. (2016) reported that absolute hippocampal volumes were significantly smaller in MD patients than in the control group *via* 3D magnetic resonance volumetric determination. A large-sample retrospective cohort study discovered that late-onset MD was related to an increased risk of all-cause dementia, including AD and Vascular Dementia (VD; Lee et al., 2021). Liu et al. (2019) found that cognitive impairment was more severe in MD patients compared to BPPV patients using the Neuropsychological Vertigo Inventory (NVI) and Cognitive Failure Questionnaire (CFQ). Despite mounting evidence showing cognitive impairment in patients with MD, studies on the specific dimensions of cognitive decline remain limited. A recent study was important, which used a broad array of measures and found that cognitive deficits in MD patients were mainly in attention, verbal learning, recognition and recall in verbal memory, recall in visual memory, visuospatial construction, and planning skills, according to comprehensive neuropsychological testing results (Eraslan Boz et al., 2023). Our results supported and validated the previous view by including a healthy control group and analyzing cognitive performance in comparison with the MD group to improve the reliability of the results. The results of our study revealed the impairment of cognitive functions, especially in memory in MD before therapy compared to HC. Furthermore, effect sizes (Cohen *d*) of group comparisons in the visuospatial/executive, attention, and orientation scores showed a medium effect. This is consistent with previous studies showing cognitive deficits in MD patients.

Although cognitive impairment in patients with MD has been well established, its related factors remain a moot question. In view of the probable mediating factors such as hearing loss and emotional stress, vestibular decline as a potential cause of cognitive decline has been overlooked or confounded to a certain extent (Lahmann et al., 2015; Dobbels et al., 2019; Smith et al., 2019; Bosmans et al., 2020; Livingston et al., 2020; Xie et al., 2022). Hence, the primary objectives of this prospective study were to evaluate cognitive function in general and different domains of MD patients before and after therapy and to determine whether the change in cognitive scores was correlated with the improvements in clinical characteristics, particularly the severity of vertigo. According to our findings, cognitive function improved significantly after therapy, particularly in memory and visuospatial/executive dimensions. It also showed improvements in orientation, attention, and language. The cognitive improvements were positively correlated with the decrease in DHI scores, especially the DHI-Physical scores, at 3 and 12 months post-treatment, which was additionally supported by the multivariate analysis. That is to say, as the severity of vertigo and related physical and functional effects reduced, cognitive performance improved. Our results preliminarily support the view that vestibular dysfunction is a potentially modifiable risk factor for cognitive decline. Effective vestibular treatments are expected to improve related cognitive decline and thus enhance the quality of life and prognosis of MD patients. However, this correlation failed to be found at 6 months post-treatment, possibly due to varying number, duration, and frequency of vertigo attacks, intervals of time since the last episode, and whether it was a typical MD episode or other ill-described chief complaints. These potential variables would be considered in future research. Moreover, we found no consistent correlation between the decrease in DHI-Emotional scores and cognitive improvement in MD patients, despite the fact that numerous studies have shown that emotional stress is relevant to cognitive decline (Lahmann et al., 2015; Smith et al., 2019; Xie et al., 2022). The lack of correlation can likely be explained by that one DHI dimension may not be sufficient for assessing all emotional dysfunction in MD patients. For another, this indirectly suggests that vestibular-related cognitive impairment is present independent of emotional disorders in MD patients.

To the best of our knowledge, similar prospective studies to evaluate cognitive function in MD patients were rare. In an important recent study, the cognitive function and severity of vertigo in patients with definite and probable MD were assessed using CFQ and DHI pre-and post-treatment (Dornhoffer et al., 2021). They reported no notable improvements in cognitive performance despite a reduction in vestibular symptoms (steady CFQ total and subfactor scores in the context of significantly reduced DHI) in definite MD patients. Although this was at variance with our results, possibly due to differences in assessment methodologies and follow-up time after therapy, both highlighted the importance of assessing and intervening in cognitive dysfunction in MD patients. On the other hand, in line with our findings, they also revealed a moderate correlation between improvements in DHI-Physical scores and CFQ false-triggering domain (the interrupted processing of cognitive and motor action sequences) scores in definite MD patients before and after therapy (Rast et al., 2009; Dornhoffer et al., 2021).

Nevertheless, no significant association between improvements in hearing loss and cognitive function was observed according to our results, which was consistent with the study conducted by Dornhoffer et al. (2021). Actually, it does not contradict the evidence that hearing loss is an independent risk factor for cognitive decline based on several possible causes. First, the average low- to midfrequency hearing thresholds merely decreased by 5–6 (dB HL) after treatment, which might be too limited to benefit cognitive function. Second, pure-tone audiometry, a subjective audiometry technique, may present about 5 (dB HL) inaccuracy with the real hearing data. What is more, from the long-term follow-up (12 months), no significant improvements were noted in hearing levels whereas hearing-related cognitive change should be a long run. It can be seen that our findings corroborated evidence of the correlation between vestibular dysfunction and cognitive impairment, regardless of hearing status. Given that MD patients in the middle and late stages often have progressive hearing loss that is challenging to recover from, the vestibular function is more likely to be a potentially modifiable risk factor for cognitive decline.

Although it was found that cognitive decline improved after effective therapy, which was related to the severity of vertigo in the present study, some limitations must be considered. First, the sample size was relatively small with a partial loss of follow-up. However, as there are few similar studies, it adds important knowledge to the current field. Second, although the DHI and MoCA are considered to be effective and precise with high retest reliability, they might be affected by participants’ current states or subjective consciousness to some extent. Additionally, although an interval of 3–6 months between cognitive tests is estimated to be adequate, the potential influence of “learning effects” cannot be overlooked. The development and validation of alternative MoCA test versions in the future could reduce “learning effects” in the repeated testing of longitudinal studies (Nasreddine and Patel, 2016; Siciliano et al., 2019). In the future, we will conduct more objective and comprehensive evaluations, such as the number, duration, and frequency of vertigo attacks, cognitive tasks in various subdomains, and neuro-electrophysiological indexes on the patients and further explore the relationships between them with larger samples.

## Data availability statement

The raw data supporting the conclusions of this article will be made available by the authors, without undue reservation.

## Ethics statement

The studies involving human participants were reviewed and approved by the Ethics Committee of Beijing Tsinghua Changgung Hospital. The patients/participants provided their written informed consent to participate in this study. Written informed consent was obtained from the individual(s) for the publication of any potentially identifiable images or data included in this article.

## Author contributions

HY conceptualized and designed the study and approved the final version of the manuscript. JZ followed up of patients, handled the acquisition of the data, performed the analysis and interpretation of the data, prepared the draft of the manuscript, and made revisions. XLi, JX, WC, JG, and XLu performed supervision and made the revisions. SL, ZG, ML, and YL offered administrative, technical, or material support. All authors contributed to the article and approved the submitted version.

## Conflict of interest

The authors declare that the research was conducted in the absence of any commercial or financial relationships that could be construed as a potential conflict of interest.

## Publisher’s note

All claims expressed in this article are solely those of the authors and do not necessarily represent those of their affiliated organizations, or those of the publisher, the editors and the reviewers. Any product that may be evaluated in this article, or claim that may be made by its manufacturer, is not guaranteed or endorsed by the publisher.
